# Informational Gene Phylogenies Do Not Support a Fourth Domain of Life for Nucleocytoplasmic Large DNA Viruses

**DOI:** 10.1371/journal.pone.0021080

**Published:** 2011-06-16

**Authors:** Tom A. Williams, T. Martin Embley, Eva Heinz

**Affiliations:** Institute for Cell and Molecular Biosciences, University of Newcastle, Newcastle upon Tyne, United Kingdom; University of British Columbia, Canada

## Abstract

Mimivirus is a nucleocytoplasmic large DNA virus (NCLDV) with a genome size (1.2 Mb) and coding capacity (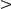 1000 genes) comparable to that of some cellular organisms. Unlike other viruses, Mimivirus and its NCLDV relatives encode homologs of broadly conserved informational genes found in Bacteria, Archaea, and Eukaryotes, raising the possibility that they could be placed on the tree of life. A recent phylogenetic analysis of these genes showed the NCLDVs emerging as a monophyletic group branching between Eukaryotes and Archaea. These trees were interpreted as evidence for an independent “fourth domain” of life that may have contributed DNA processing genes to the ancestral eukaryote. However, the analysis of ancient evolutionary events is challenging, and tree reconstruction is susceptible to bias resulting from non-phylogenetic signals in the data. These include compositional heterogeneity and homoplasy, which can lead to the spurious grouping of compositionally-similar or fast-evolving sequences. Here, we show that these informational gene alignments contain both significant compositional heterogeneity and homoplasy, which were not adequately modelled in the original analysis. When we use more realistic evolutionary models that better fit the data, the resulting trees are unable to reject a simple null hypothesis in which these informational genes, like many other NCLDV genes, were acquired by horizontal transfer from eukaryotic hosts. Our results suggest that a fourth domain is not required to explain the available sequence data.

## Introduction

Resolving the tree of life is among the most interesting and challenging questions in evolutionary biology. Although it is widely held that the Archaea, Bacteria and Eukarya form three distinct domains of life, two competing hypotheses place the Eukaryotes either as a sister taxon to the Archaea–the so-called 3 domains tree [1]–or emerging from within a paraphyletic Archaea as the sister group of the Crenarchaeotes or Eocyta–the so-called eocyte hypothesis [2].

These debates, however, have focused on the relationships among cellular lineages, excluding viruses. This approach has been justified on both philosophical and pragmatic grounds. Philosophically, it has been argued that viruses are selfish elements that lack their own metabolism and are therefore more comparable to transposons than to independent lifeforms [3]. More practically, the small genomes of viruses did not contain enough information to reliably position them on the tree of life [4]. The latter argument was weakened by the discovery of Mimivirus, a nucleocytoplasmic large DNA virus (NCLDV) with a genome of unprecedented size (1.2Mb) and coding capacity (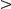 1,000 ORFs), exceeding that of many cellular organisms [5], [6]. In an initial phylogenetic analysis, Mimivirus emerged from the branch joining Archaea and Eukaryotes, suggesting that it might represent a distinct fourth domain of life [6]. The division of the tree of life into domains is difficult, because the existing classification is based on patterns of similarity in ribosomal RNAs that are not found in viruses [1]. Nonetheless, the position of Mimivirus as an outgroup to Eukaryotes has concrete biological consequences. In particular, this result motivated the proposal that the lineage formed by Mimivirus and its NCLDV relatives might have contributed DNA processing genes to the ancestral eukaryote [7]. However, a subsequent re-analysis of the dataset used in [6] indicated that the position of Mimivirus was an artifact: the genes that were concatenated to build the phylogeny had been acquired by horizontal transfer (HGT) from different sources, resulting in an inconsistent phylogenetic signal that placed them as the outgroup to Eukaryotes [8]. Further analyses demonstrated that NCLDVs have obtained many genes by horizontal transfer from across the three cellular domains [9], [10].

In principle, extensive HGT should not preclude the placement of NCLDVs on the tree of life. Prokaryotic phylogeny is famously obscured by HGT [11] to the extent that rings [12] or networks [13] arguably represent evolutionary history better than trees. With this in mind, multi-domain phylogenies have focused on the small core of genes that are present across all genomes being compared, and which are thought to be resistant to HGT [14]–[16]. This list comprises a subset of genes involved in DNA replication, transcription, and translation–the so-called “informational” genes–which may more closely represent the evolutionary history of the lineages that carry them [17]. Does the same logic apply to NCLDVs, or were their informational genes acquired by HGT along with much of the rest of their genomes [9], [10]?

Recently, Boyer and colleagues [18] presented new evidence for the “4 domains” hypothesis based on a phylogenetic analysis of 12 informational genes involved in nucleotide biosynthesis, DNA replication, transcription and translation. This study included sequences from Bacteria, Archaea, NCLDVs, and Eukaryotes. Trees based on 8 of the 12 informational genes either suggested that the NCLDVs were polyphyletic or were unable to provide compelling support for the 4 domains hypothesis, because they contained sequences from only one family of NCLDVs (the Mimiviridae). However, in four cases – the topologies inferred from RNA Polymerase II (RNAP2), Transcription Factor II Beta (TFIIB), Flap Endonuclease (FEN), and Proliferating Cell Nuclear Antigen (PCNA) – the trees show the viruses emerging as a monophyletic group from the branch linking the Archaea and Eukaryotes. This result lead Boyer et al. [18] to hypothesize that the NCLDVs represented a “4th domain” of life whereby the core genome of the NCLDVs was as old as the three cellular domains, and to reiterate the idea that this group of viruses might have contributed information processing genes to the ancestral Eukaryote.

The divergence of a “4th domain” would represent a very early event during evolutionary history, but successful reconstruction of ancient evolutionary events is notoriously difficult due to variation in evolutionary rate and base composition among the lineages being compared [19]. Among-lineage rate variation can lead to an artifact known as long branch attraction (LBA), in which fast-evolving lineages are grouped with each other or with a highly-divergent outgroup [20]. LBA occurs when parallel (convergent) substitutions in fast-evolving sequences are misinterpreted as shared derived characters, and can result in serious topological errors. For instance, the placement of the Microsporidia (fast-evolving relatives of the Fungi) at the base of the Eukaryotic tree rather than with Fungi [21], [22] and the exclusion of Acoelomorph flatworms from the deuterostomes [23], have both been attributed to LBA. Although not as well studied, compositional heterogeneity is known to cause distantly-related lineages to group together in trees due to covergent sequence composition [24]. An example is the clustering of thermophilic bacteria in trees based upon ribosomal RNA sequences due to convergence to high G+C content [25].

A number of strategies for dealing with LBA and compositional heterogeneity among sequences have been proposed. Fast-evolving sites can be removed from the analysis [26], the data can be recoded using a reduced alphabet [27], [28], or LogDet distances can be used [29], [30]. Alternatively, these features can be incorporated into the evolutionary models used to infer trees under maximum likelihood or Bayesian methods. Several such models have been developed in recent years, and have been shown to improve both model fit and topological estimation on problematic datasets [23], [31]–[35].

The analyses of Boyer et al. [18] used traditional evolutionary models (JTT [36] and WAG [37]) of the type that can be sensitive to LBA and/or compositional heterogeneity [22], [32]. Moreover, previous analyses of deep phylogeny have revealed that substantial differences in evolutionary rates and composition affect molecular sequences sampled from the three cellular domains [15]. Combined with the fast evolutionary rates and compositional bias (see below) of viral sequences, these considerations raise the possibility that the basal position of the NCLDVs in published phylogenies [18] might be influenced by model misspecification.

To investigate this possibility, we re-analysed the data from [18], evaluating the fit of the models used with respect to sequence saturation, homoplasy and compositional heterogeneity. Our results showed that the original models did not adequately account for these features of the data, so we explored the fit of a range of more complex models that were developed to better account for one or more of these features [23], [31]–[33], [35]. Using these models, we could not reject the hypothesis that the informational genes of NCLDV, like many of the other genes of this group [9], [10], have been acquired by horizontal transfer from donors within the eukaryotic domain. Our results suggest that invoking an ancient “4th domain” for NCLDV, or a special primordial role for NCLDV in the formation of Eukaryotes, are not needed to explain the available molecular sequence data for this group of viruses.

## Materials and Methods

### Sequences and alignments

Protein sequences for RNAP2, TFIIB, FEN and PCNA were obtained from NCBI GenBank and from the Joint Genome Institute using the accession numbers from [18]. In several cases, these numbers did not appear to correspond to the sequences used in the original analysis. In these cases, we performed BLASTP searches against the original genome using the most closely related available sequence as a query. When performing the analysis (January/February 2011), the novel viral sequences reported in [18] were not available from GenBank. The absence of these sequences are unlikely to have affected our analyses substantially, because only two of the new sequences were homologs of the five proteins we re-analyze here. Further, these sequences were obtained from Courdovirus and Moumouvirus, both of which are members of the Mimiviridae. Since this group is already represented in the relevant alignment (TFIIB), and the monophyly of the group is not in question, their absence is unlikely to affect our results.

Each set of homologs was aligned with MUSCLE [38] and T-COFFEE [39], and a consensus alignment was built with META-COFFEE [40]. These alignments were processed following the protocol of [18], in which Gblocks [41] was used to select well-aligned columns from the initial alignment. The parameters of Gblocks were varied in order to produce an alignment as close as possible to that used to produce the original “4 domains” trees, with similarity assessed by the number of alignment columns selected by Gblocks. For one of the four genes (RNAP2), we were able to produce an alignment of exactly the same length as in [18] (272 aligned positions). In the other three cases, we obtained alignments with similar, but not identical, numbers of positions (TFIIB: 162 used in this study vs. 155 in the original study; FEN: 215 vs. 304; PCNA: 178 vs 174). Since even lax Gblocks parameters can result in stringent selection criteria, we investigated whether we could include more positions in our analyses by manually editing the initial alignments and removing poorly-aligning regions “by eye”. We performed two tests to evaluate the reliability of these “manual” alignments relative to the original Gblocks ones. First, we used the Heads-or-Tails method [42], which calculates the discrepency between alignments generated from sequences written in the forward and reverse directions. Since these alignments should be identical under ideal conditions, the extent of the difference between them provides a measure of the ambiguity in the data that cannot be resolved by the alignment procedure. This analysis showed that the manual alignments were substantially less reliable than the Gblocks ones (see Table S1). Second, we compared the number of different amino acids per site between each pair of alignments using non-parametric Mann-Whitney *U* tests. This approach indicated that the manual alignments were significantly less conserved (P 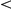 0.05 in all cases). Since these analyses suggested that our manual alignments contained more noise and ambiguity than our Gblocks alignments, we used the more conservative Gblocks alignments in all of our subsequent phylogenetic analyses.

### Phylogenetic analysis

We used ProtTest [43] to select the best-fitting substitution matrix for our alignments of each of the four genes. In each case, the LG matrix [44] was chosen over the JTT [36] and WAG [37] matrices used in the original analysis by both the AIC and BIC criteria (see Table S1). We nonetheless performed our analyses with all three matrices in order to compare our results to those of Boyer et al [18]. The better fit of the LG matrix was further supported by maximum likelihood analyses, which gave the highest likelihood for the tree obtained with this matrix (see Table S1). Maximum likelihood trees were generated using RAxML [45], [46] with 1000 rapid bootstrap inferences followed by a thorough ML search under the gamma model and an ML estimate of the alpha-parameter.

Bayesian analyses were performed with p4 [32] and PhyloBayes [47]. We used these packages because they implement more complex evolutionary models that better account for compositional heterogeneity and site-specific biochemical diversity, respectively. The node-discrete compositional heterogeneity (NDCH) model discussed below is implemented in p4, while the UL3, CAT10 and CAT60 models are implemented in PhyloBayes.

In p4, the “autoTune” option was used to optimize parameter acceptance rates before starting the runs, and for each analysis, two independent runs were perfomed. Individual runs were performed for 2 million generations running 4 chains in parallel (3 heated), sampling every 2500 generations and generating 10 checkpoints. The runs were performed under the gamma model with 4 categories, where the alpha-parameter was estimated from the data during the run. For each sampling point, data was simulated and used for later compositional heterogeneity analyses. Convergence was assessed by comparing the average standard deviation of splits between two runs. One quarter of each run was discarded as burn-in for all analyses as well as for generation of the consensus tree.

### Modelling compositional heterogeneity

For each of the alignments, one analysis was performed under the JTT, the WAG and the LG rate exchange matrix using one compositional vector, estimated from the data during the run. Additional vectors were added iteratively to our runs under the LG matrix until an adequate model fit was achieved, as assessed with a compositional Chi-Square test. To perform this test, the Chi-Square value for compositional heterogeneity from the original alignment was compared to a distribution of Chi-Square values calculated from data simulated during the run. A tail-area probability test was performed, and the model was considered to fit based on compositional heterogeneity when the value obtained from the alignment fell within the central 95% of the values estimated from the simulated data.

As a complementary approach to reduce both compositional heterogeneity and substitutional saturation, we recoded our alignments into the six Dayhoff groups of amino acids with similar chemical properties [48]. Dayhoff recoding was performed as described previously [15], [49], and Dayhoff-recoded datasets were used for tree calculations as described above, with a rate exchange matrix estimated from the data during the run and with one, two or three base compositional vectors for each dataset depending on whether the simulated data fit the original value for compositional heterogeneity.

### Modelling site-specific biochemical diversity

For each alignment, calculations were performed with the JTT, WAG and LG matrix as well as the mixture models UL3 [34], CAT10 and CAT60. These mixture models represent an advance on single-matrix models because they better account for the site-specific biochemical features of the evolutionary process, either with a mixture of matrices (UL3) or of equilibrium frequency profiles (CAT10/60). We used these empirical CAT models, in which the set of frequency profiles have been estimated on the HSSP alignment database, in preference to the related CAT [31] or CAT-BP [50] models because of the limited amount of data available in single-gene analyses [35]. Previous work has shown that they almost always improve model fit [34] and, in the case of the CAT models, are less susceptible to long branch attraction artifacts compared to single-matrix models such as JTT, WAG and LG [33]. As with our other analyses, we used 4 gamma rate categories with an alpha parameter estimated from the data during the run. Two independent PhyloBayes runs were executed for each alignment and model. The chains were stopped at regular intervals and convergence was assessed by comparing discrepancies in bipartition (split) frequencies and summary variables (including mean posterior log likelihood, tree length, and the alpha parameter for across-site rate variation) between the two runs. For bipartitions, the difference measure used was the maximum discrepancy in bipartition posterior probabilities, while for the other variables it was twice the difference of the means between the two runs divided by the sum of their standard deviations. After discarding 1/4 of each run as burn-in, points were sampled every 10 cycles, and runs were considered to have converged when the maximum discrepancies were 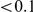, with the effective size of each summary variable 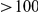, as recommended by the authors (http://www.phylobayes.org). If a pair of runs converged, these samples were combined to obtain a consensus tree. Posterior predictive simulations [51] were performed on the chain that had run longest, using the same sampling strategy. The observed value of site-specific biochemical diversity was calculated from the data and compared to a distribution simulated under the model. As with the tests for compositional heterogeneity, the test was considered to have failed when the observed value fell outside the central 95% of the simulated distribution.

## Results and Discussion

### Single-matrix models do not fit the data

An initial comparison of the available single-matrix evolutionary models using ProtTest [43] demonstrated that the LG matrix was the best-fitting model under both the AIC and BIC criteria, in preference to the JTT and WAG matrices used in [18]. The assessment of relative fit allows the selection of the best model from a set of candidates, but whilst this shows that a model is superior to other choices, it does not guarantee that the chosen model actually fits the data well. To investigate the fit of the models used to analyse the data with respect to site-specific biochemical features and compositional heterogeneity, we used posterior predictive simulations [51] in a Bayesian framework implemented in the phylogenetics packages p4 [32] and PhyloBayes [47]. Each point sampled from the MCMC chain was used to simulate data on which the statistic of interest (in p4, Chi-Square test for compositional heterogeneity [32]; in PhyloBayes, site-specific biochemical diversity [33]) was evaluated. If the model fits (that is, the model used in the course of the calculation could have generated the data), then the value of the statistic calculated on the real data should fall within the distribution of simulated values. Here, we consider such a test to have failed if the real value does not fall within with the central 95% of the distribution of simulated values (i.e., p 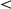 0.05).

Our analyses revealed that none of the single-matrix evolutionary models – neither the JTT and WAG models used by Boyer et al. [18], nor the better-fitting LG model – adequately fit the data, as all of them underestimate the level of compositional heterogeneity and overestimate the site-specific biochemical diversity present in all four alignments (see Table 1). Both of these problems can cause errors in tree inference [24], [26], raising the possibility that the support for the “4 domains” hypothesis obtained from these proteins might be influenced by model misspecification.

**Table 1:** Single-matrix models underestimate the level of compositional heterogeneity and overestimate the site-specific biochemical diversity of the informational gene alignments.

Test	RNAP2 (JTT)	TFIIB (WAG)	FEN (WAG)	PCNA (WAG)
**Compositional heterogeneity**	794.89+/−74.23 (969.23)	397.04+/−36.63 (533.11)	399.1+/−33.24 (659.46)	547.47+/−41.02 (828.28)
**Site specific diversity**	7.07+/−0.13 (5.79)	7.72+/−0.17 (7.01)	8.05+/−0.15 (6.8)	8.67+/−0.16 (7.75)

Predicted distributions (means and standard deviations) and observed value (in parenthesis) of the test statistic. All differences are significant at P 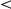 0.05. The models are those used in [18]; the LG model also fails all the tests for all four genes (see Table S1).

The trees recovered by our analyses using the JTT, WAG or LG models share features in common with the previously published trees [18] for these data, but also show important differences. Thus, we obtained the same 4 domains tree for RNAP2 using the JTT model as did Boyer et al [18], but with much lower support in our analyses for the critical node supporting the monophyly of the NCLDVs; an SH-like local support value [52] of 0.22 versus the 0.82 of [18] (see Supporting Information S1). Our ML and Bayesian analyses under the JTT and WAG models produced 4-domain majority rule consensus trees for RNAP2 and FEN, but in the trees for TFIIB and PCNA the NCLDVs either emerged from within the eukaryotic domain, or the relationships among the eukaryotic and viral sequences could not be resolved at posterior probability 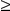 0.5).

### Improving fit with mixture models

To investigate if the lack of model fit might be affecting the tree topologies recovered, we reanalysed the data using models that attempt to better account for site-specific biochemical features and compositional heterogeneity. The results of these analyses are summarised in Table 2, with representative trees illustrated in Figures 1, 2, 3 and 4 (all other trees passing our model tests are provided as Figures S1, S2, S3, S4, S5, S6, S7, and S8).

**Figure 1:**
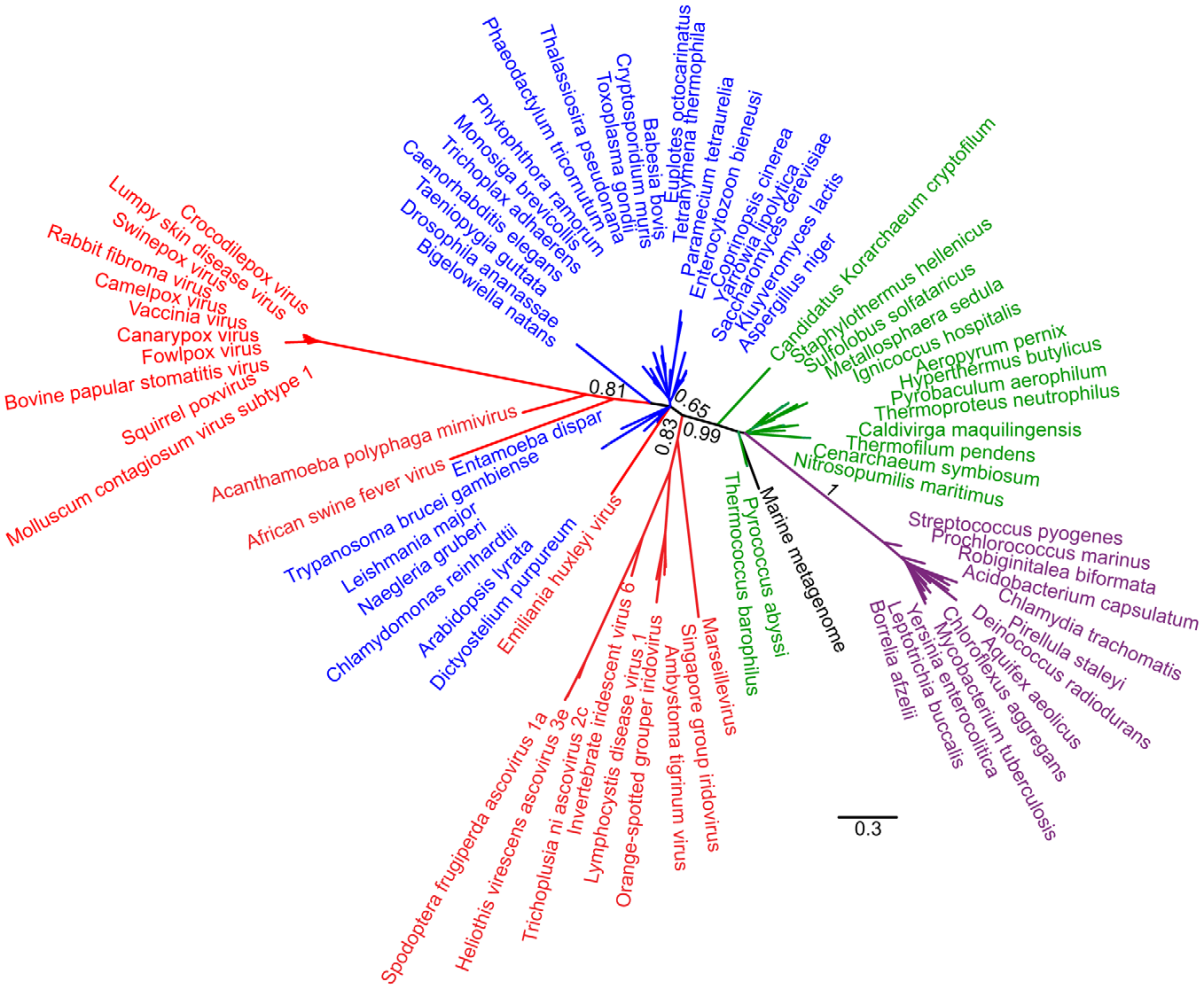
Unrooted phylogeny of RNAP2 based on Bayesian analysis of 80 sequences of 272 amino acid positions performed with PhyloBayes under the CAT60 model. Detailed parameters are given in the Materials and Methods section. Assuming that the root of the tree lies outside the viruses and eukaryotes, the NCLDV sequences (red) are not monophyletic but form three groups, one branch located between the archaeal (green) and the eukaryotic (blue) sequences, one branch emerging from within the eukaryotes, and one branch comprising the *Emiliana huxleyi* virus. Bacterial sequences are shown in purple, and metagenomic sequences of unknown organismal origin are shown in black. Branch support shown represents posterior probabilities, bar represents 0.3 substitutions per site.

**Figure 2:**
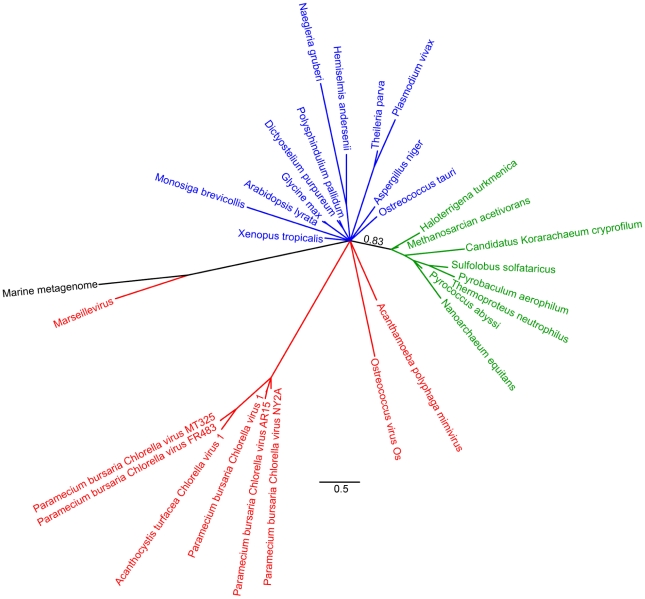
Unrooted phylogeny of TFIIB based on Bayesian analysis of 30 sequences of 162 amino acid positions performed with PhyloBayes under the CAT60 model. Detailed parameters are given in the Materials and Methods section. This tree shows a polytomy in which the relationships among the the different eukaryotic groups and NCLDV lineages are not resolved at posterior probabilities 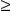 0.5. This lack of resolution beyond the eukaryote/prokaryote split is typical of the topologies recovered for this gene under the models that passed our tests. Archaeal sequences are shown in green, the black sequence represents a metagenomic sequence of unknown organismal origin. The indicated branch support values are posterior probabilities, and the bar represents 0.3 substitutions per site.

**Figure 3:**
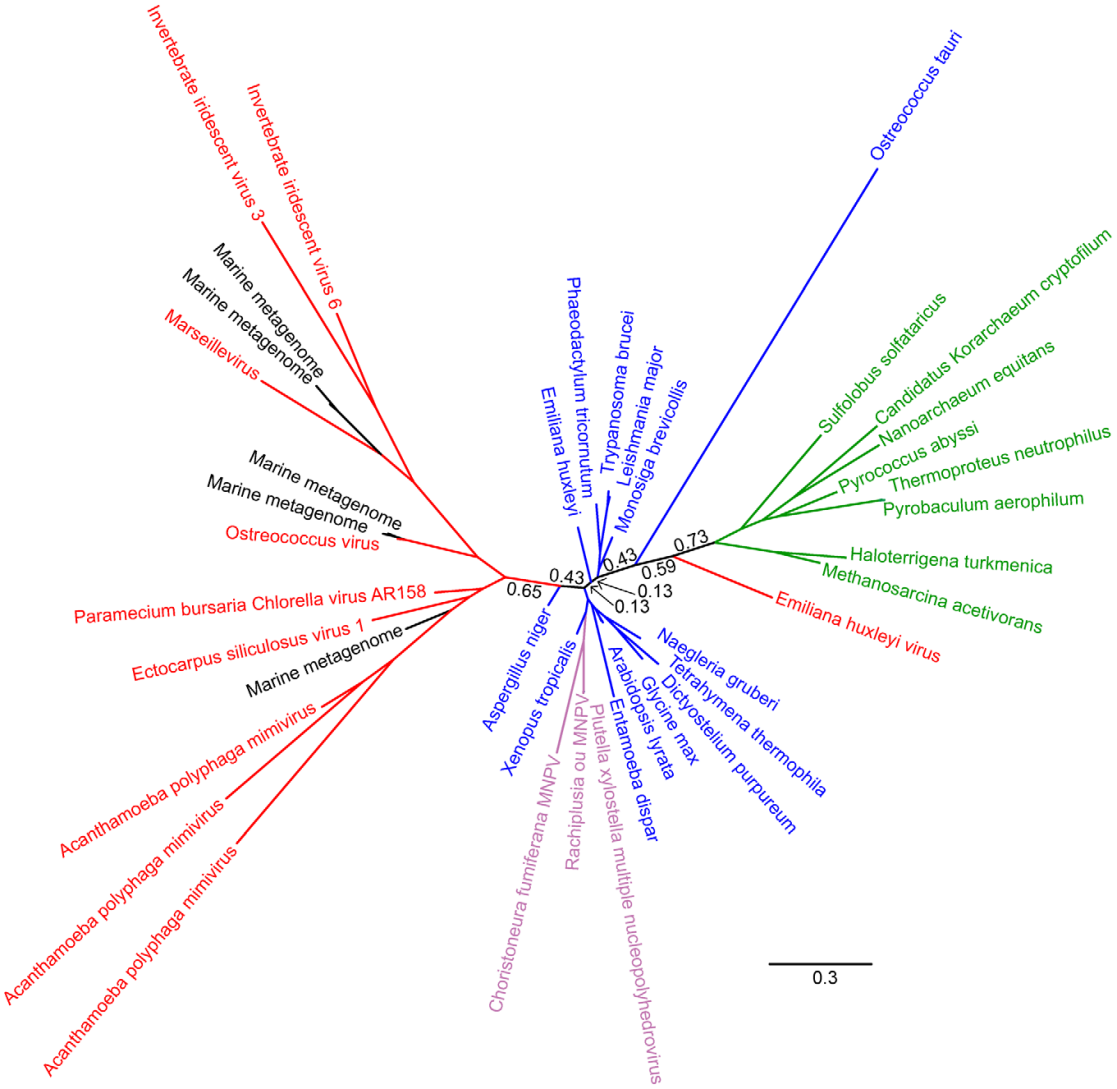
Unrooted phylogeny of PCNA based on Bayesian analysis of 40 sequences of 178 Dayhoff-recoded amino acid positions performed with p4 with an additional base composition vector. Detailed parameters are given in the Materials and Methods section. The NCLDV sequences (red) and metagenomic sequences (black) emerge as a single group from within the eukaryotes (with the exception of the *Emiliana huxleyi* virus). Archaeal sequences are in green. The indicated branch support values are posterior probabilities, and the bar represents 0.3 substitutions per site.

**Figure 4:**
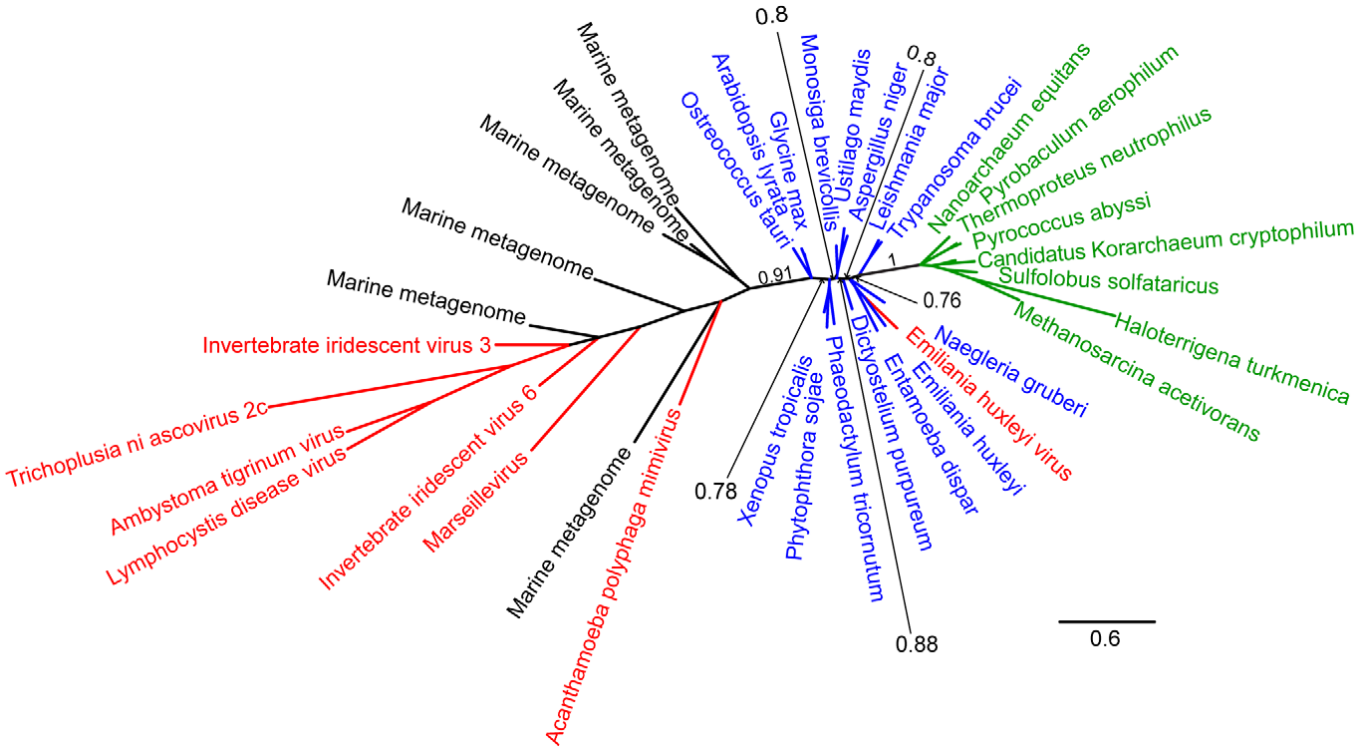
Unrooted phylogeny of FEN based on Bayesian analysis of 37 sequences of 215 amino acid positions performed with PhyloBayes under the CAT60 model. Detailed parameters are given in the Materials and Methods section. The NCLDV sequences (red) and metagenomic sequences (black) emerge as a single group from within the eukaryotes (blue), with the exclusion of the *Emiliana huxleyi* virus. Archaeal sequences are in green. The indicated branch support values are posterior probabilities, and the bar represents 0.6 substitutions per site. Black sequences represent metagenomic sequences of unknown organismal origin.

**Table 2:** Support for the 4th domain of life hypothesis [18] from analyses of informational genes using different models.

Test	RNAP2	TFIIB	PCNA	FEN
**JTT**	Yes(f)	No(f)	No(f)	No(f)
**WAG**	Yes(f)	No(f)	No(f)	Yes(f)
**LG**	Yes(f)	No(f)	No(f)	Yes(f)
**LG+1**	Yes(p)	No(f)	No(f)	Yes(f)
**LG+2**	n.a.	No(p)	No(f)	Yes(f)
**Dayhoff**	No(p)	No(p)	No(f)	No(f)
**Dayhoff+1**	n.a.	n.a.	No(p)	No(p)
**UL3**	No(f)	No(p)	No(p)	No(f)
**CAT10**	No(f)	No(p)	No(f)	Yes(f)
**CAT60**	No(p)	No(p)	No(f)	No(p)

“Yes” denotes a “4-domains” topology sensu Boyer et al [18] in which the NCLDVs emerge as a monophyletic group branching between Archaea and Eukaryotes; in cases where we report no support, the NCLDVs emerge from within the eukaryotic domain, or the relationships among viruses and Eukaryotes are not resolved at posterior probability 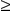 0.5. (f) indicates a model mis-specification and thus failure of the respective test with regards to compositional heterogeneity, biochemical diversity, or both; (p) indicates that both tests were passed. In case of a compositional fit, no further vectors were added to the respective model, indicated by ‘n.a.’ (not analyzed). The addition of 2, 3 and 4 Dayhoff vectors led to a similar result with regards to topology, but a failure of the ChiSquare test.

To incorporate the varying amino acid composition identified in our alignments, we used the node-discrete compositional heterogeneity (NDCH) model [32], which allows composition to change over the tree, combined with the JTT, WAG and LG substitution matrices. Due to the better fit of the LG matrix based on the AIC and BIC measures (see Table S1), we performed analyses with the LG matrix with added base-composition vectors for all of the proteins. Although adding additional compositional vectors markedly improved model fit with respect to compositional heterogeneity for all of the data sets, we were unable to fit the model to the data for the PCNA and FEN alignments at our stringency criterion (see Table 2). Therefore, we also recoded these data into the six biochemically-similar Dayhoff groups [48]. This recoding has been shown to reduce both substitutional saturation and compositional heterogeneity [27], [49], [53], and it allows an alignment-specific substitution matrix to be estimated from the data. This recoding allowed us to fit the model with respect to compositional heterogeneity for PCNA and FEN, emphasising the high level of heterogeneity in the original un-recoded data (see Table 2).

Six trees passed our test for compositional heterogeneity – two each for RNAP2 and TFIIB (NDCH combined with either the LG matrix plus composition vectors, or Dayhoff recoding of the data), and one each for PCNA and FEN (under the NDCH model with Dayhoff recoding and one additional composition vector). The only split in these trees that was consistently strongly supported (posterior probability 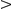
0.95) was between the viruses and eukaryotes on one side, and the prokaryotes on the other. Since we are predominantly interested in the relationship between the NCLDVs and eukaryotes we therefore used the prokaryotes as an outgroup to orientate the subsequent discussion of our tree topologies. Considering the consensus topologies obtained from the 6 analyses, 4 trees (for TFIIB, PCNA and FEN) show the NCLDV weakly branching from within the eukaryotes in a poorly resolved, in terms of posterior probabilities, radiation. In the tree produced for RNAP2 using the NDCH model with 1 additional composition vector, we recovered all of the NCLDV sequences, with one exception, as a weakly supported (posterior probability 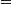 0.57) clade at the base of the eukaryotes. However, the viral branches were the longest in the tree – a consistent pattern across all our analyses, as well as in the trees reported in [18]. When we recoded the RNAP2 amino acid data into Dayhoff classes, an approach that can sometimes mitigate long branch attraction [27], [49], the NCLDV sequences branched within the eukaryotic radiation. In summary, our analyses designed to mitigate the effects of compositional heterogeneity among sequences, which is clearly a feature of these data, generally recovered weakly supported topologies that are consistent with the NCLDV receiving their genes from within the eukaryotic domain, rather than providing support for a 4th domain of life sensu Boyer et al [18].

To further investigate the effect of possible model misspecification on tree topologies, we used three mixture models: UL3 [34], CAT10, and CAT60 [35] that were designed to better accommodate site-specific patterns of protein evolution. In real proteins, different residues experience different functional constraints, so that only a subset of the 20 possible amino acids may be present at any one site [54]. The mixture models allow different sites to be fit by three different substitution matrices (UL3) or a set of 10 or 60 equilibrium frequency profiles (CAT10 and CAT60). Since most of the site specific profiles comprise only a small number of amino acids, site-specific diversities generated under these models tend to be lower (in agreement with real data) than with traditional models. Potential sequence saturation and homoplasy are also better modelled, because the probability of convergent evolution at any particular site under the model is recognised as being higher than if 20 amino acids were represented. As a result, these mixture models are reported to be more resistant to LBA than traditional evolutionary models [33].

We obtained six trees that passed our posterior predictive test for site-specific biochemical diversity: one each for RNAP2, PCNA, and FEN, and three for TFIIB (we obtained more trees for TFIIB because all three of the models tested passed in this case; see Table 2). As we observed when modelling compositional heterogeneity, the strongest signal in these trees (posterior probability 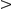
0.95) is the split between the prokaryotes on the one hand, and the viruses and eukaryotes on the other; so as before we use the prokaryotes as an outgroup to orientate our discussion of the relationships between NCLDV and eukaryotes. None of the trees are otherwise strongly supported and therefore they do not provide compelling evidence for a separate 4th domain [18], for the NCLDV viruses investigated. In the cases of TFIIB (Figure 2) and PCNA, the NCLDV and eukaryotic sequences formed an unresolved polytomy suggesting that there is insufficient signal in the data to resolve the position of NCLDVs relative to eukaryotes at posterior probabilities 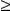 0.5. In the FEN phylogeny (Figure 3), the viruses emerged as a monophyletic group from within the eukaryotes with weak support, while in the RNAP2 phylogeny the virus sequences were split into two groups; one group at the base of eukaryotes and the other from within the eukaryotic radiation (Figure 4).

Our results suggest that the sequences investigated are unable to strongly resolve the position of NCLDV relative to eukaryotes even when we better model compositional heterogeneity and site specific biochemical diversity. One possibility is that the data contains conflicting convergent signals (i.e. homoplasies) that are able to compete with the signal for phylogeny [50]. Models that underestimate the amount of homoplasy in the data may be particularly susceptible to misinterpreting homoplasy as synapomorphy and may therefore inflate support for spurious relationships including long branch attraction (LBA). To investigate whether excess homoplasy might be affecting our analyses, we compared observed and predicted levels of homoplasy in the 4 alignments under each of the models used. Because the true evolutionary history of the alignment, and therefore the real number of homoplasies, is not known, it must be estimated under the model [50] by stochastic substitution mapping [55]. For each model and alignment, a distribution of the test statistic was calculated from simulated substitutional histories that were conditional on the data, compared to a distribution calculated from unconstrained substitution mappings. The agreement between the two provided a measure of how well each of the models anticipated homoplasy.

**Figure 5:**
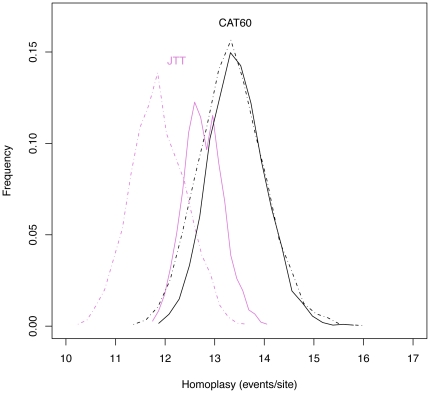
Observed (solid lines) and predicted (dashed lines) numbers of homoplasic events per site for the RNAP2 alignment under the JTT (purple) and CAT60 (black) models. This case illustrates the pattern seen for three of the four genes (see Table 3): under the CAT model, which predicts substantially higher levels of homoplasy, there is good agreement (P = 0.38) between the observed and predicted distributions. The JTT model predicts significantly less homoplasy than it observes (P = 0.018), and the means of both distributions are lower than under CAT60. This suggests that CAT60 anticipates, and is better able to account for, higher levels of homoplasy in the data.

As Table 3 indicates, the UL3 and CAT60 models consistently predict higher levels of homoplasy than the single-matrix models investigated here and previously used by Boyer et al [18]. Further, while there are no significant differences between the observed and predicted distributions for the mixture models (P
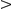
0.05), the single-matrix models “fail” this test for three of the four genes (RNAP2, PCNA, and FEN). The mean and variances of the observed and predicted distributions are provided for all models in Table S1; a graphical comparison of the distributions under the JTT (used in [18]) and CAT60 models is provided for the RNAP2 gene in Figure 5. These results indeed suggest that the JTT, WAG and LG models do not adequately anticipate the level of homoplasy in the alignments: making them more likely to misinterpret homoplasy as synapomorphy and potentially more susceptible to LBA.

**Table 3:** Homoplasy (mean predicted homoplasic events/site +/− variance) in each gene under the homogeneous models used in [18] (JTT for RNAP2, WAG for the others) and the UL3 and CAT60 mixture models.

Model	RNAP2	TFIIB	PCNA	FEN
**Boyer et al. (2010)**	11.94+/−0.55 (0.018)	9.54+/−0.64 (0.061)	17.18+/−0.92 (0.015)	10.76+/−0.52 (0.0028)
**UL3**	14.23+/−0.72 (0.165)	16.76+/−1.22 (0.43)	26.04+/−1.64 (0.05)	19.49+/−1.02 (0.71)
**CAT60**	13.49+/−0.67 (0.38)	16.95+/−1.98 (0.67)	23.31+/−1.68 (0.66)	16.34+/−1.18 (0.59)

The mixture models predict greater levels of homoplasy, in line with the observed values (the P-value for each test is given in brackets; see the main text for a discussion of how this comparison is performed). A complete version of this table is available in Table S1.

### Better-fitting models do not support the “4-domains” hypothesis

In our analyses, we have compared support for two hypotheses to explain the origin(s) of NCLDV informational genes for RNAP2, PCNA, FEN and TFIIB. The first is that they were obtained by the same mechanism that appears adequate to explain the origin(s) of other genes now residing on NCLDV genomes; namely by horizontal transfer from within the eukaryotic or bacterial domains [9], [10]. The alternative hypothesis is that of Boyer et al [18], whereby these genes are taken to have descended vertically from an ancestral NCLDV lineage that branched as an independent “fourth domain” between archaea and eukaryotes. This second hypothesis also underpins suggestions that NCLDV may have contributed genes during the formation of eukaryotes [7], [18].

A complicating factor in our analysis was the substantial non-phylogenetic signal present in all four of the genes we analysed. Compositional heterogeneity, site specific diversity and homoplasy are all known to influence phylogenetic inference when inadequately modelled [15], and they are all present in the data sets analysed by ourselves and Boyer et al [18]. Single-matrix models such as JTT, WAG and LG recover “4 domains” topologies for some of the proteins, but these models fail to adequately account for either changing amino acid compositions, homoplasy or site-specific biochemical diversity. When these features were accommodated by better models, the support for an NCLDV 4th domain effectively disappeared: the NCLDV sequences emerged from within the eukaryotes, or the relationships between eukaryotes and viruses were not resolved. Thus a 4th domain for NCLDV viruses in the sense proposed in Boyer et al [18], or a role for NCLDVs in eukaryotic origins [7], [18], do not appear either to be supported by, or necessary to explain, these particular data.

Part of the rationale given by Boyer et al. [18] for using RNAP2, TFIIB, PCNA and FEN to infer the evolutionary history of the NCLDV lineage is that, in cellular organisms, these genes form part of a widely-conserved “genealogy-defining core” of genes. These are informational genes that encode proteins performing essential “housekeeping” functions and are thought to be more resistant to horizontal transfer than the rest of the genome [17]. Although RNAP2, TFIIB, FEN and PCNA are completely conserved in eukaryotes and archaea, they appear to have a patchier distribution among viruses. Although Mimivirus and Marseillevirus possess all four of the genes, the other NCLDVs possess only a subset of these genes (see the trees of [18] and our Figures 1, 2, 3 and 4). In other words there appears to have been extensive secondary losses (or these genes were never present in some lineages) across the NCLDV tree. This pattern demonstrates that there is no equivalent “genealogy-defining core” for all NCLDV and suggests that the functional constraints on these genes in NCLDVs is not as strong as in cellular lifeforms.

## Supporting Information

Figure S1 **Unrooted phylogeny of RNAP2 based on Bayesian analysis of 80 sequences of 272 Dayhoff-recoded amino acid positions performed with p4.** Detailed parameters are given in the Materials and Methods section.
(PDF)

Figure S2 **Unrooted phylogeny of RNAP2 based on Bayesian analysis of 80 sequences of 272 amino acid positions performed with p4 under the LG model with one additional base composition vector.** Detailed parameters are given in the Materials and Methods section.
(PDF)

Figure S3 **Unrooted phylogeny of TFIIB based on Bayesian analysis of 30 sequences of 162 Dayhoff-recoded amino acid positions performed with p4.** Detailed parameters are given in the Materials and Methods section.
(PDF)

Figure S4 **Unrooted phylogeny of TFIIB based on Bayesian analysis of 30 sequences of 162 amino acid positions performed with p4 under the LG model with two additional base composition vectors.** Detailed parameters are given in the Materials and Methods section.
(PDF)

Figure S5 **Unrooted phylogeny of TFIIB based on Bayesian analysis of 30 sequences of 162 amino acid positions performed with PhyloBayes under the UL3 model.** Detailed parameters are given in the Materials and Methods section.
(PDF)

Figure S6 **Unrooted phylogeny of TFIIB based on Bayesian analysis of 30 sequences of 162 amino acid positions performed with PhyloBayes under the CAT10 model.** Detailed parameters are given in the Materials and Methods section.
(PDF)

Figure S7 **Unrooted phylogeny of PCNA based on Bayesian analysis of 40 sequences of 178 amino acid positions performed with PhyloBayes under the UL3 model.** Detailed parameters are given in the Materials and Methods section.
(PDF)

Figure S8 **Unrooted phylogeny of FEN based on Bayesian analysis of 37 sequences of 215 Dayhoff-recoded amino acid positions performed with p4 with one additional base composition vector.** Detailed parameters are given in the Materials and Methods section.
(PDF)

Table S1 **Additional statistical information.** Alignment quality comparisons, model test results, tree topologies and likelihoods from our phylogenetic analyses.
(XLS)

Supporting Information S1 This archive contains the raw data used in our analyses. The alns subfolder contains the sequences and alignments used. FASTA headers contain the accession numbers for the protein sequences; in some cases, these are truncated in the PHYLIP files to accommodate the restrictions of the phylogenetics packages. The trees subfolder contains the trees in Newick or Nexus format for all of the analyses (including runs that failed one or more of our model tests). The files follow the naming convention gene_model; e.g. tf2b_ul3.tre denotes the tree of the TFIIB sequences under the UL3 mixture model. The result of running the FastTree analysis of Boyer et al. on our RNAP2 alignment is called rnap2_fasttree.tre in the root of this directory.
(RAR)
