# Supplementary figures and images for: Informational Gene Phylogenies Do Not Support a Fourth Domain of Life for Nucleocytoplasmic Large DNA Viruses
Source: PLoS One. 2011 Jun 16;6(6):e21080. doi: 10.1371/journal.pone.0021080 (PMC3116878; doi:10.1371/journal.pone.0021080)

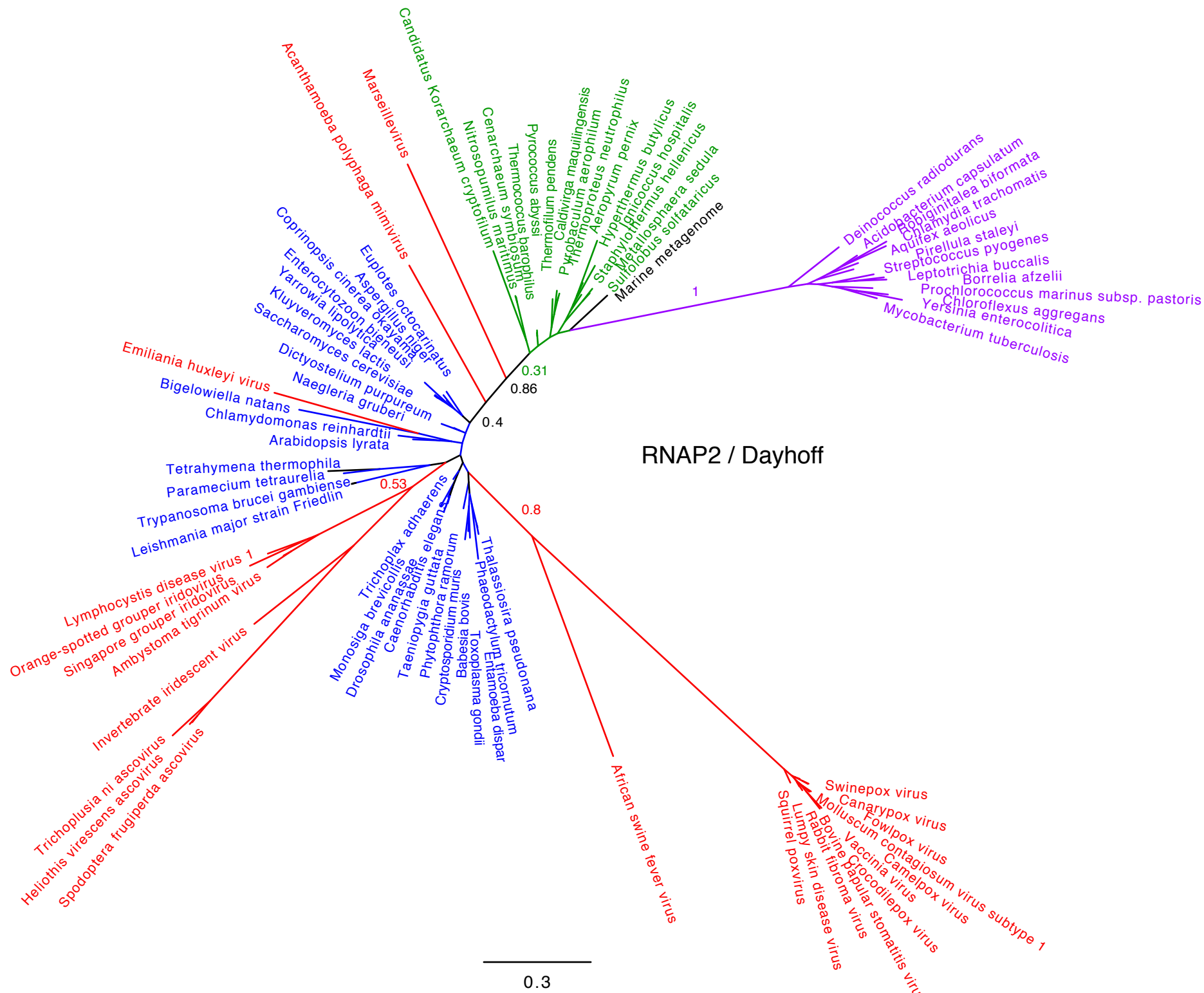

Supplement: Figure S1 — Unrooted phylogeny of RNAP2 based on Bayesian analysis of 80 sequences of 272 Dayhoff-recoded amino acid positions performed with p4. Detailed parameters are given in the Materials and Methods section. (PDF) [file pone.0021080.s001.pdf]

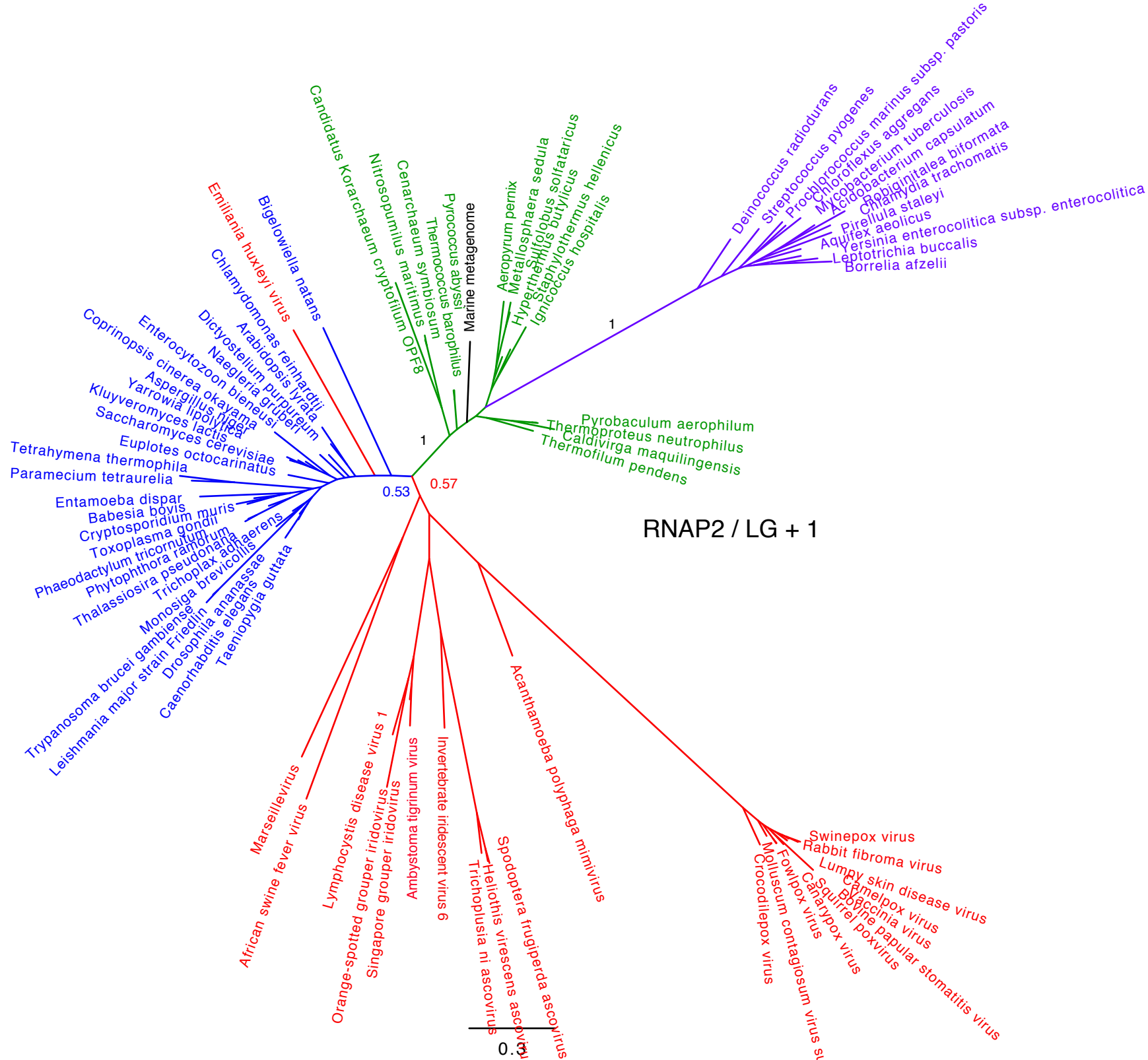

Supplement: Figure S2 — Unrooted phylogeny of RNAP2 based on Bayesian analysis of 80 sequences of 272 amino acid positions performed with p4 under the LG model with one additional base composition vector. Detailed parameters are given in the Materials and Methods section. (PDF) [file pone.0021080.s002.pdf]

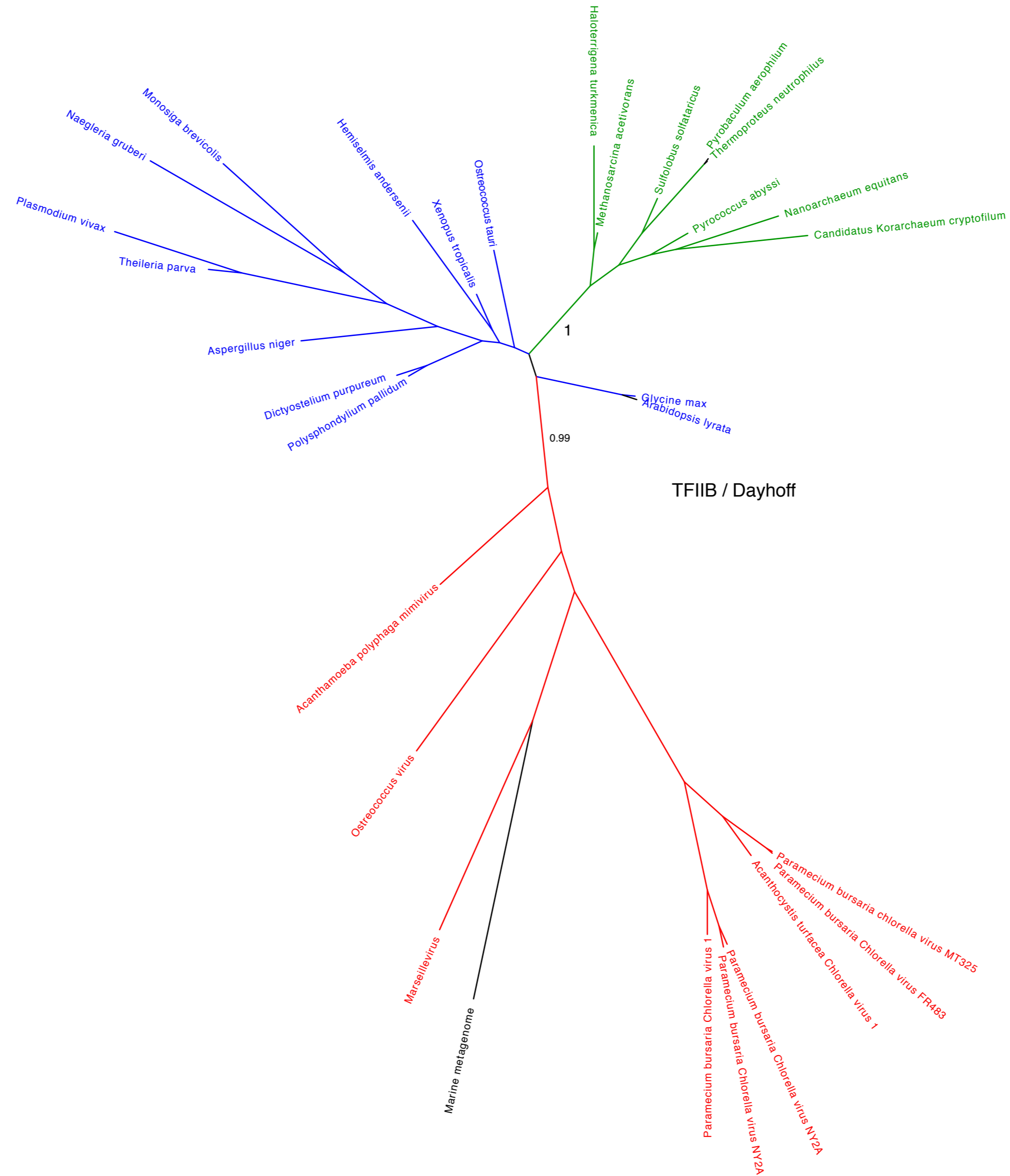

Supplement: Figure S3 — Unrooted phylogeny of TFIIB based on Bayesian analysis of 30 sequences of 162 Dayhoff-recoded amino acid positions performed with p4. Detailed parameters are given in the Materials and Methods section. (PDF) [file pone.0021080.s003.pdf]

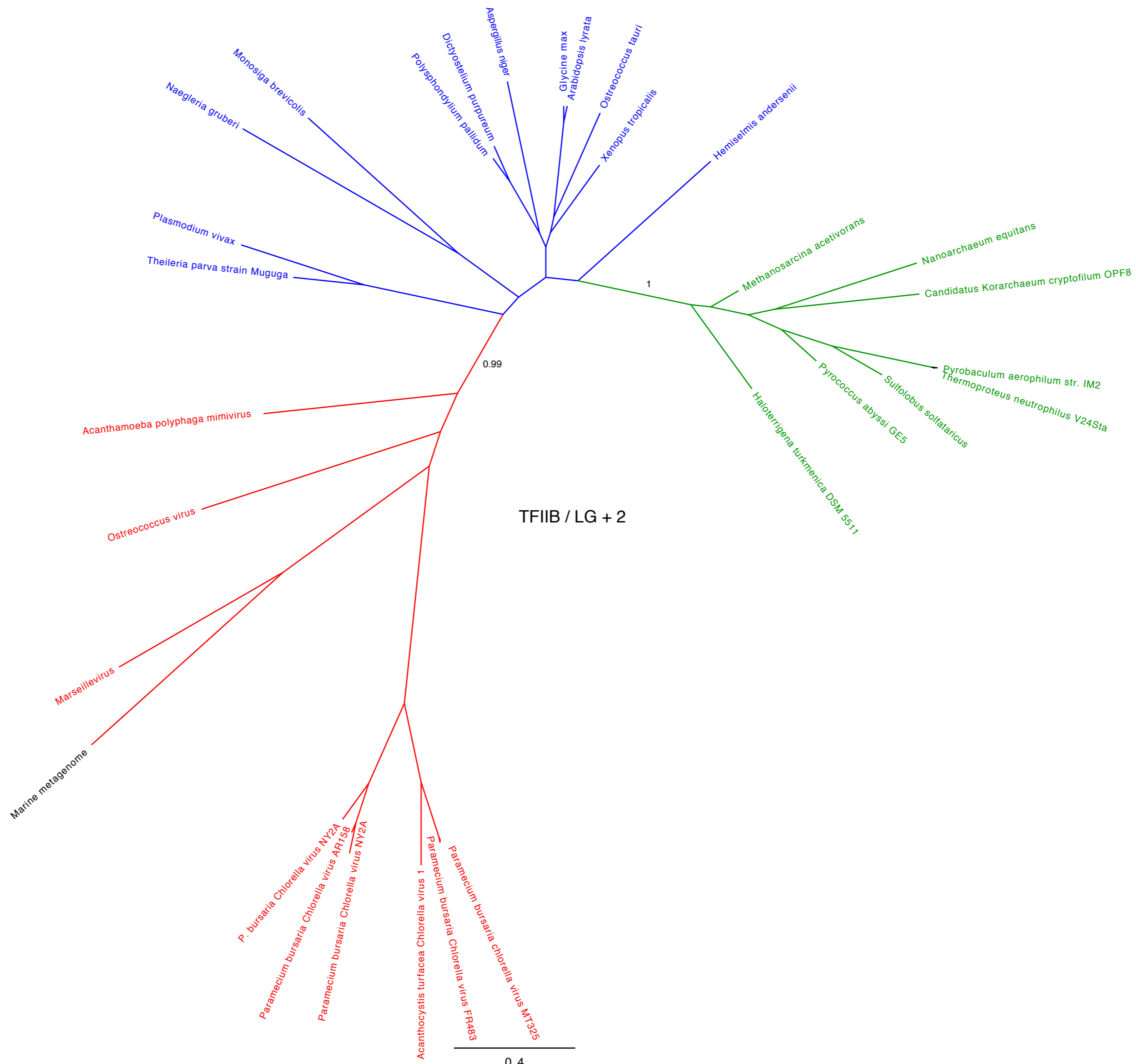

Supplement: Figure S4 — Unrooted phylogeny of TFIIB based on Bayesian analysis of 30 sequences of 162 amino acid positions performed with p4 under the LG model with two additional base composition vectors. Detailed parameters are given in the Materials and Methods section. (PDF) [file pone.0021080.s004.pdf]

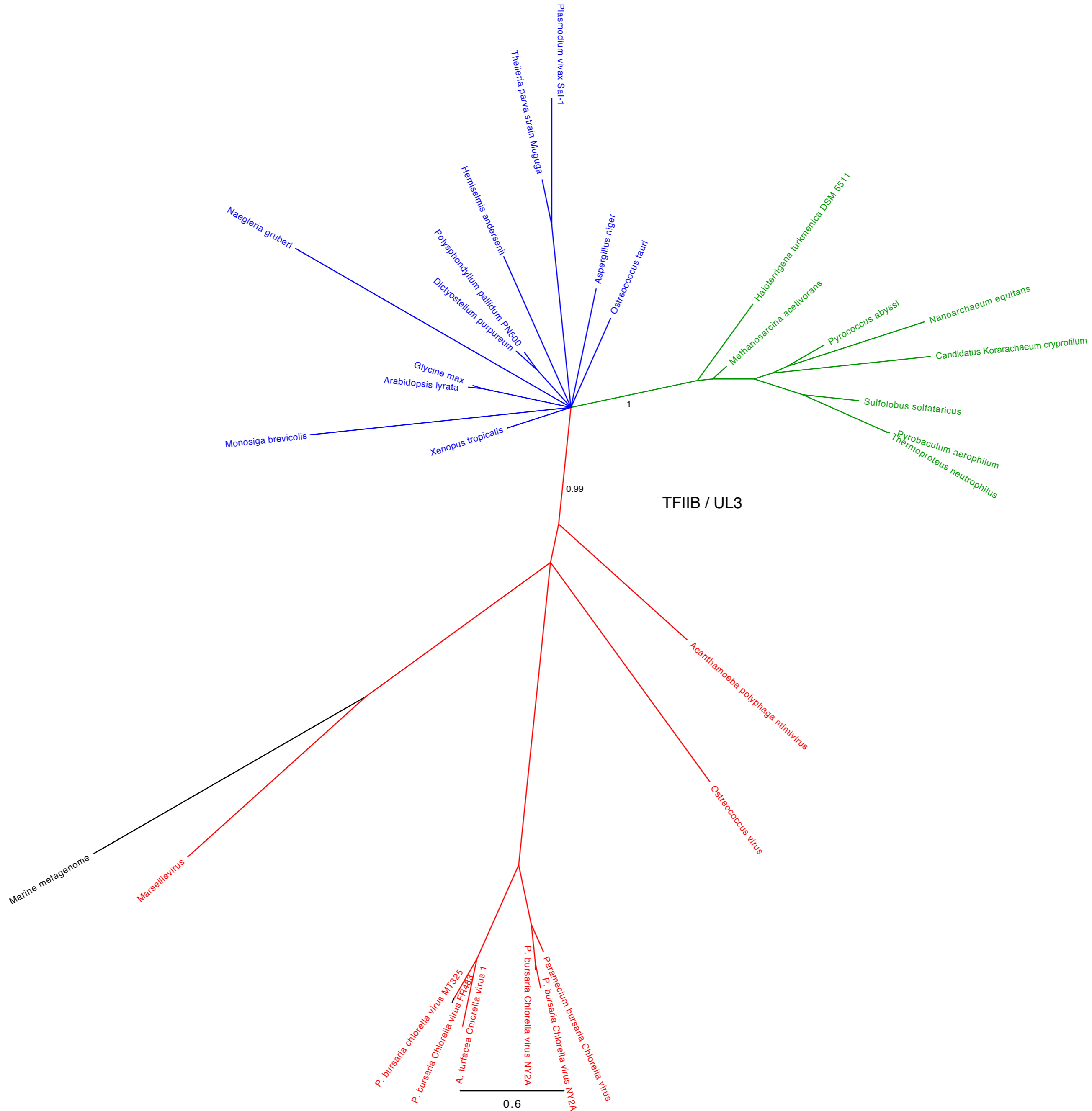

Supplement: Figure S5 — Unrooted phylogeny of TFIIB based on Bayesian analysis of 30 sequences of 162 amino acid positions performed with PhyloBayes under the UL3 model. Detailed parameters are given in the Materials and Methods section. (PDF) [file pone.0021080.s005.pdf]

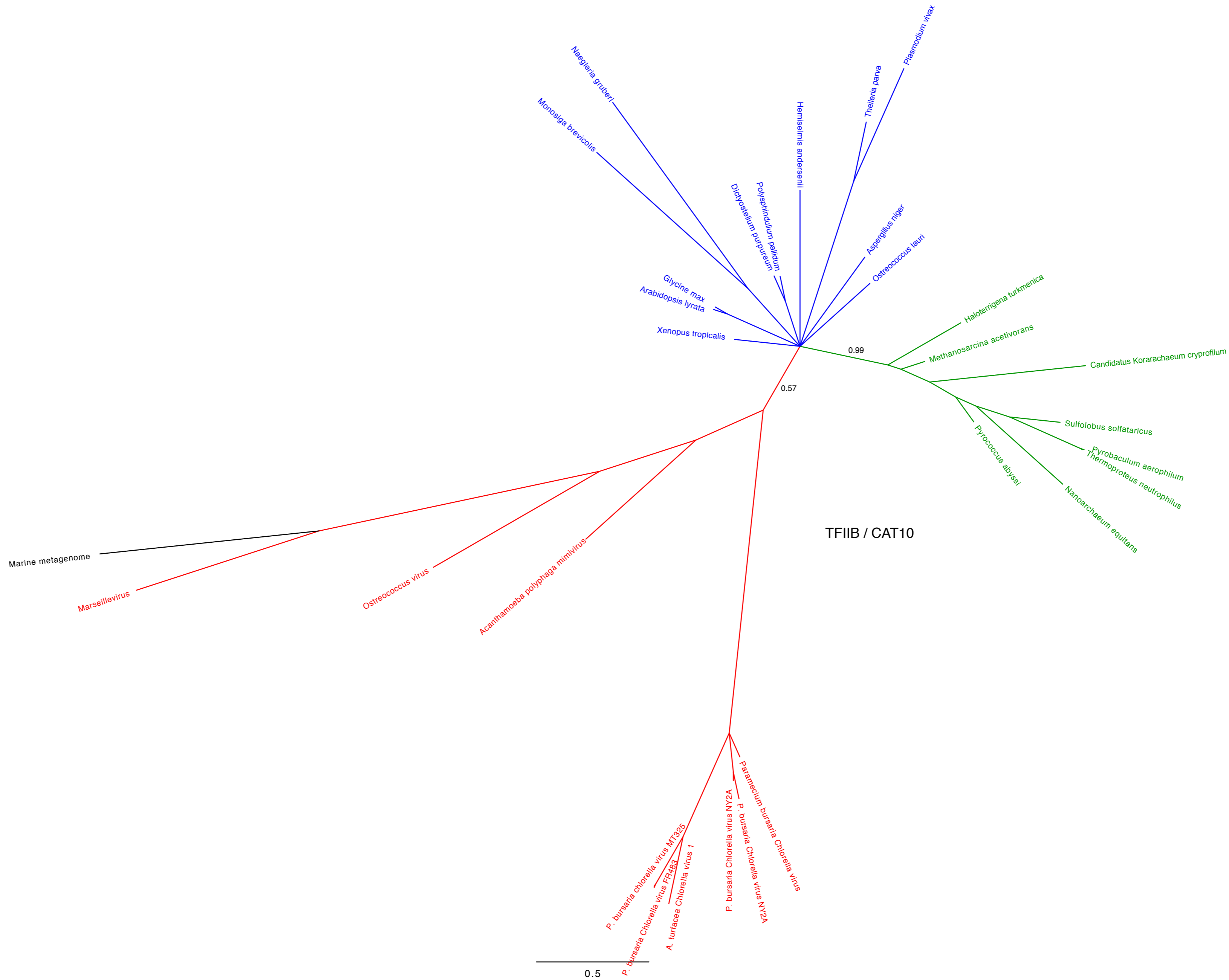

Supplement: Figure S6 — Unrooted phylogeny of TFIIB based on Bayesian analysis of 30 sequences of 162 amino acid positions performed with PhyloBayes under the CAT10 model. Detailed parameters are given in the Materials and Methods section. (PDF) [file pone.0021080.s006.pdf]

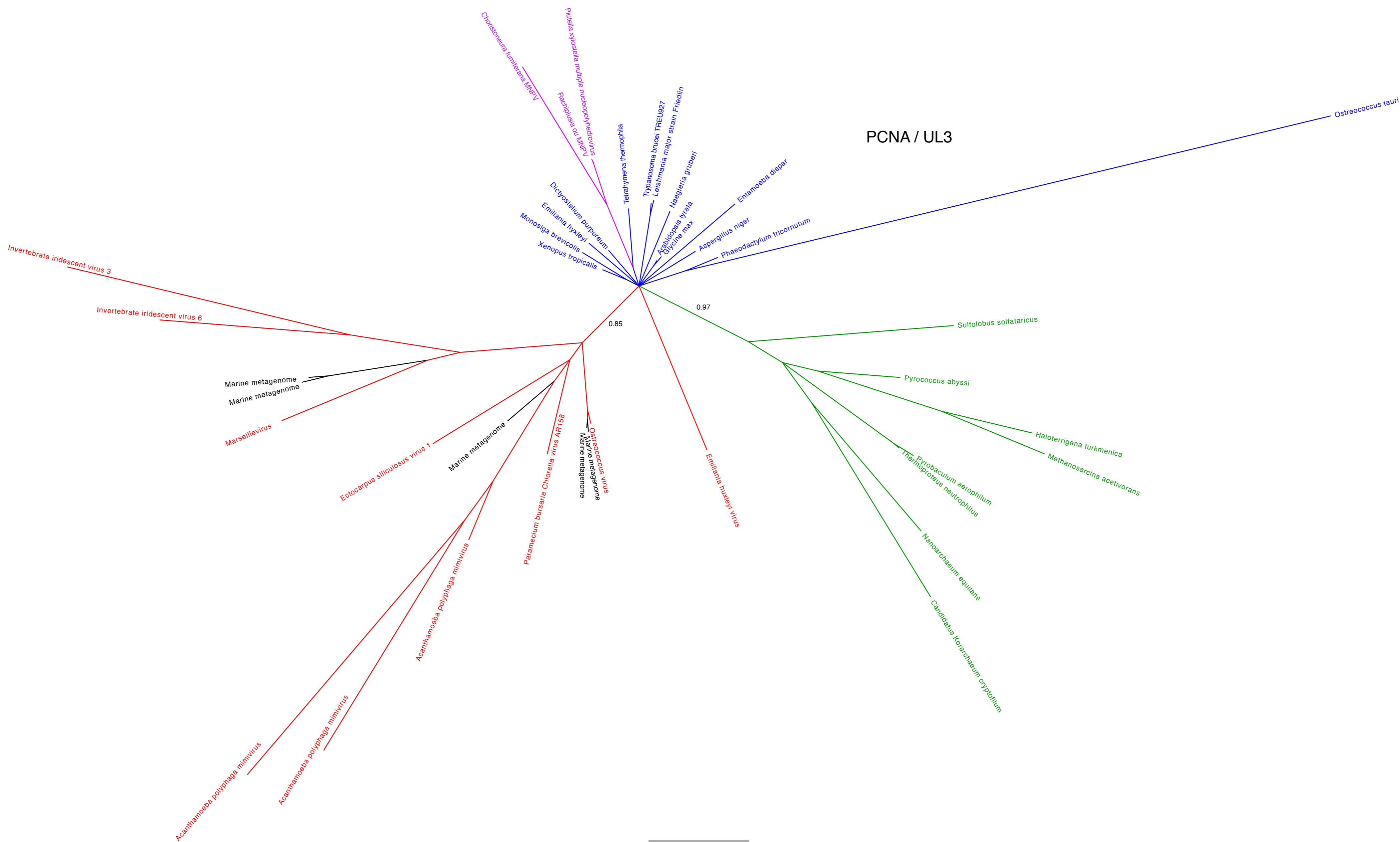

Supplement: Figure S7 — Unrooted phylogeny of PCNA based on Bayesian analysis of 40 sequences of 178 amino acid positions performed with PhyloBayes under the UL3 model. Detailed parameters are given in the Materials and Methods section. (PDF) [file pone.0021080.s007.pdf]

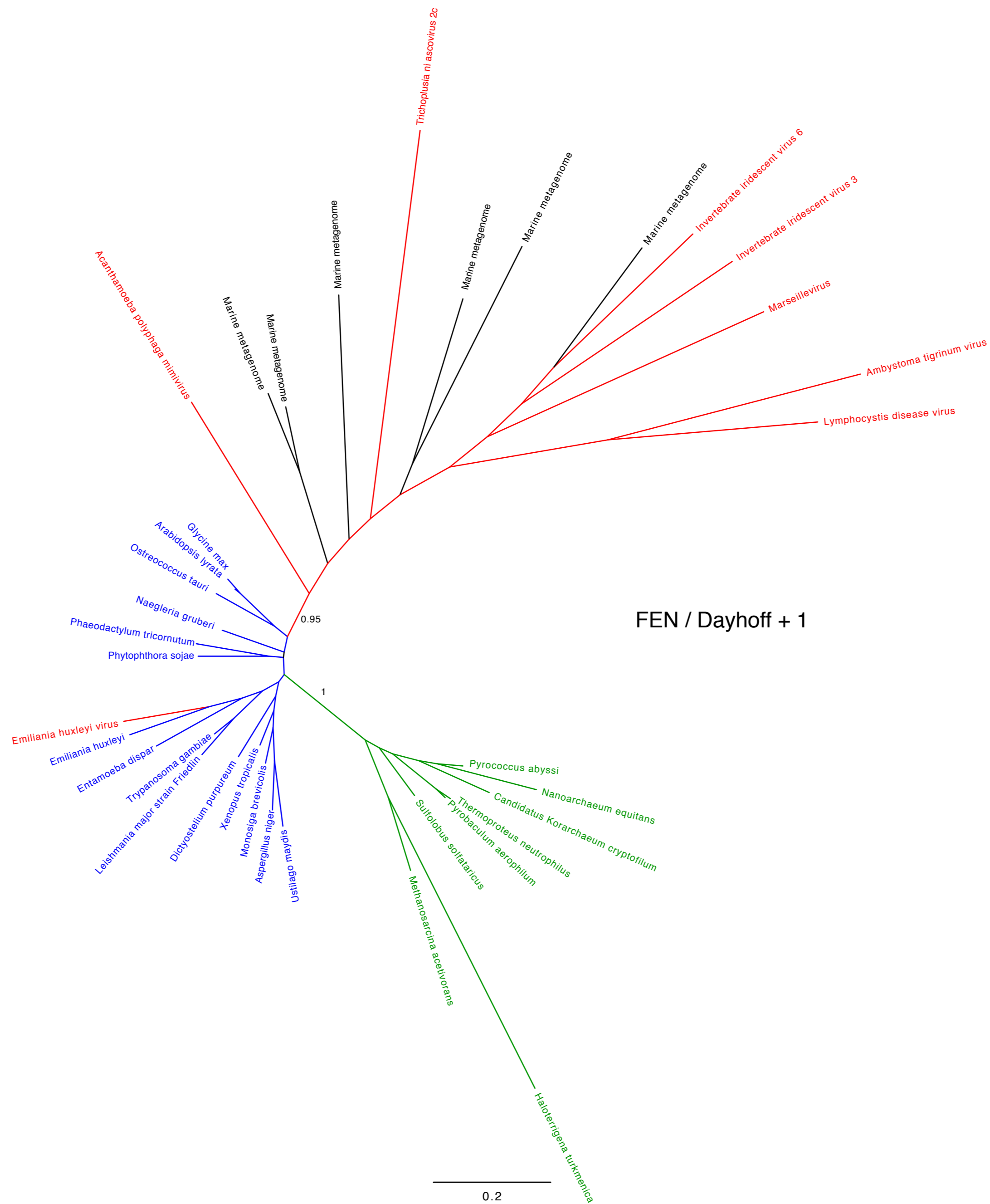

Supplement: Figure S8 — Unrooted phylogeny of FEN based on Bayesian analysis of 37 sequences of 215 Dayhoff-recoded amino acid positions performed with p4 with one additional base composition vector. Detailed parameters are given in the Materials and Methods section. (PDF) [file pone.0021080.s008.pdf]
